# Medical News and Miscellany

**Published:** 1893-02

**Authors:** 


### Medical News and Miscellany.

The Cooper Medical College, of San Francisco, has adopted a four years course of instruction.—*Ex.*

The English historian, Freeman, who always opposed vaccination, has just died of smallpox.—*Ex.*

Dr. E. J. Ward, of Waxahachie, so well known to the Texas profession, died in Waxahachie December 17.

A bill is now before the Texas Legislature to castrate criminals who are sentenced to the penitentiary for rape.

Dr. A. P. Baldwin, of Tyler, Texas, has just returned from the New York Polyclinic, where he took a three months' special course.

Dr. V. A. Howeth, so long a resident of Gainesville, Texas, and lately President of Cook County Medical Society, has removed to Pomona, California.

There is a vacancy on the Board of Regents, caused by the election of Judge Simkins to the Supreme Court. It would be interesting to know who will be the appointee.

Married.—In Austin, Texas, February 8th inst., Dr. H. L. Hilgartner to Miss Belle Palm, both of Austin. Dr. Hilgartner is oculist to the State Institute for the Blind, vice Dr. T. J. Tyner, resigned.

A Tribute to Villemin.—The Le Conte prize of $10,000 has been awarded to the heirs of Professor Villemin, by the Paris Academy of Science, for his researches in the infectiousness of tuberculosis.—*Ex.*

The Samuel D. Gross prize of $1000 will be awarded in June, 1893, for the best original essay of not more than 150 printed pages, upon some subject is surgical pathology. For particulars address Dr. J. Ewing Mears, 1429 Walnut street, Philadelphia.

Married.—December 22d, at Ledbetter, Texas, Dr. W. H. Walker to Miss Julia Gillespie, both of Ledbetter, Rev. Q. T. Simpson, officiating. In our last issue we announced the removal of Dr. Walker to Oakland, but we had not then heard of his marriage.

**A number** [illegible] experienced prescription druggist and chemist, speaks Spanish and English, desires a situation in some city drug store. Satisfactory credentials as to character and capacity. Address "Druggist," care Dr. Daniel, this office.

---

**Dr. Keiller** will gratefully receive, pay expenses on, and acknowledge specimens bearing on midwifery and diseases of women, or still born fœtuses, if sent to him at the University, Galveston, in good condition. Points of interest will be reported, and the specimens placed in the College Museum.

---

**Dr. Bruce P. McVey**, late of Ella, Brazoria county, and known to the JOURNAL'S readers through his contributions to its pages, has removed to Mumford, Robertson county, and formed a copartnership with Dr. C. D. Cearnal, an old practitioner of that section and a staunch supporter of the Red Back.

---

**Dr. E. G Cochran** has resigned his position as Chief Surgeon of the Mexican Central railroad hospital at Tampico and will return to Texas. The Doctor will go to Marshal for the present; meantime will select a location and resume practice. His successor at Tampico is Dr. A. D. Galleger, from Pennsylvania.

---

**Dr. E. G. Nicholson**, of Del Rio, whose serious illness at San Antonio the JOURNAL mentioned last month, died at Mineral Wells, Texas, on the 3d of February inst. His malady was Bright's disease. Dr. Nicholson was the most prominent physician in Val Verde county, and a popular member of the State Medical Association.

---

**The Quarantine** Bill, as amended in the U. S. Senate, was passed on the 6th inst, It gives the President power to suspend immigration if necessary to prevent the introduction of disease, and enlarges the powers of the Marine Hospital service, but *does not* give the Supervising Surgeon General or the Secretary of the Treasury power to absorb State quarantines, as was feared would be the case; State and National quarantines co-operate.

---

THE HEALTH RESTORATIVE Co's bill for $12,50 due this JOURNAL for advertising, a part of last year, can be bought very cheap by any one having confidence in that concern. We are unable to collect it. Notwithstanding this they had the audacity to ask a renewal of contract (through an agent) and we declined to accept it. The large patronage of the JOURNAL has been selected with such discriminating care that in nearly eight years the JOURNAL has lost only two bills,—this is the third,—by bad debts.

**A Mere Trifle.**—One of our Texas contemporaries tells of a doctor who, in jumping from a moving train "broke his leg at the knee, and the shoulder was badly bruised," and adds, "his injuries are not serious." This reminds us of the lady who says that during the first shocks of the earthquake at Charleston she was terribly alarmed, and her husband, to pacify her, said with emphasis, oh, don't be alarmed ! it is *nothing but an earthquake!*

---

It is stated that both the Rush Medical College and the College of Physicians and Surgeons, of Chicago, have offered to give up their entire property to the Chicago University, and the Faculties to resign unconditionally, in order that a medical depertment may be organized on a level with other schools in this already wonderfully well organized institution. The sum of a million dollars is in sight for the endowment of such a medical department.—*Ex.*

---

**Dr. Frank O. Hoyt**, the founder of the *St. Joseph (Mo.) Medical Herald*, and more recently Assistant Physician to the Missouri State Lunatic Asylum, has been appointed Superintendent of the Iowa (State) Hospital for the Insane, at Clarinda, Iowa. Dr. Hoyt is quite a young man, but has made great reputation already as a physician and neurologist. The JOURNAL extends its congratulations to the Doctor; it likes to see such evidences of appreciation of merit, and of the *strong endeavor* on the part of the young men to *get there.*

---

**In addition** to the contributions by the Faculty of the Medical Department of the University of Texas, to the several "departments" in the JOURNAL, we are pleased to announce that Prof. Seth M. Morris, M. D., of the chair of Chemistry, will take charge of the Department of Chemistry, Toxocology and Urinology, and will, each second month, contribnte notes of interest to the general practitioner.

We intend to make the TEXAS MEDICAL JOURNAL second to none in America in point of scientific interest. Subscriptions can begin at any time.

---

**There's no Place Like Texas.**—Dr. Wm. Caston, a long time resident of Corsicana, Texas, removed some years ago to Washington, and settled at Spokane Falls, where he did a fine practice in his specialty, eye and ear. He left there, however, last year, and went to Denver, Colorado. The JOURNAL is in receipt of a letter now, advising that the doctor, on account of a "complication of circumstances which rendered it necessary, for the comfort and pleasure of his family," has returned to Corsicana, his old home. The doctor will re-join the State Medical Assiciation at its next meeting, and contribute a paper to the Opthalmological Section.

**Jacobi vs. Peroxide Hydrogen.**—The JOURNAL has received a pamphlet containing Dr. A. Jacobi's paper read before the Pediatric Society, in which he indulges in very severe remarks against the medicinal peroxide of hydrogen, as sold by the Drevet Chemical Company (Marchand), and also several articles from eminent physicians, as strongly endorsing the preparation.

The JOURNAL is an advocate of fair play; and from a careful reading of the pamphlet, we are disposed to think that Dr. Jacobi is unnecessarily severe in his strictures. Mr. Marchand has introduced, by way of rebuttal, the testimony of eminent clinicians, based upon an extensive experience with the peroxide, and to our way of thinking, Dr. Jacobi is answered. There is no medicine, or preparation, but that if abused, or in unskillful hands, may disappoint one's expectations, or do harm. The value of Marchand's medicinal peroxide is well established.

